# Total syntheses of surugamides and thioamycolamides toward understanding their biosynthesis

**DOI:** 10.1007/s11418-022-01662-x

**Published:** 2022-11-08

**Authors:** Takefumi Kuranaga

**Affiliations:** Division of Bioinformatics and Chemical Genomics, Department of System Chemotherapy and Molecular Sciences, Graduate School of Pharmaceutical Sciences, Kyoto University, Yoshida, Sakyo-ku, Kyoto 606-8501 Japan

**Keywords:** Natural products, Total synthesis, Biosynthesis, Structural determination, Peptides

## Abstract

Peptidic natural products have received much attention as potential drug leads, and biosynthetic studies of peptidic natural products have contributed to the field of natural product chemistry over the past several decades. However, the key biosynthetic intermediates are generally not isolated from natural sources, and this can hamper a detailed analysis of biosynthesis. Furthermore, reported unusual structures, which are targets for biosynthetic studies, are sometimes the results of structural misassignments. Chemical synthesis techniques are imperative in solving these problems. This review focuses on the chemical syntheses of surugamides and thioamycolamides toward understanding their biosynthesis. These studies can provide the key biosynthetic intermediates that can reveal the biosynthetic pathways and/or true structures of these natural products.

**Graphical abstract:**

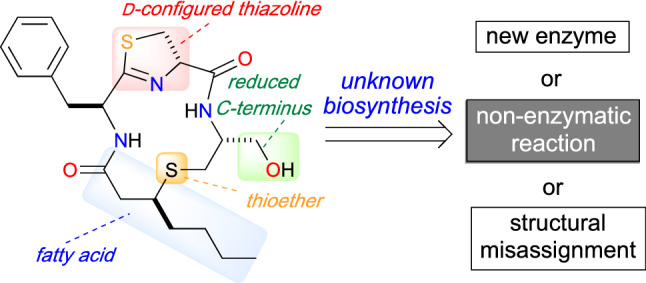

## Introduction

Small peptides (molecular weight ranging from 500 to 6000 Daltons), which have physicochemical and biological properties intermediate between those of antibodies and small molecules [1], have received much attention as ideal drug leads. The specific bioactivity of a peptide drug lead is a result of its particular structures [2], and an unprecedented structure would be constructed by a novel biosynthetic pathway.

Over the last few decades, biosynthetic studies of peptidic natural products have played a pivotal role in the discovery of new drug leads. Homology searching of biosynthetic enzymes and/or genetic manipulation of the biosynthetic processes can lead to the discovery of cryptic natural products [3]. However, the key biosynthetic intermediates, namely the substrates of the unusual enzymes, are generally not isolated from the natural sources, and this hampers the mechanistic analysis of the new biosynthetic enzymes. Additionally, the reported structures of new natural products with unusual features, which are fascinating targets for biosynthetic studies, are sometimes turned out to be the products of structural misassignments [4]. Chemical synthesis techniques are imperative for solving these problems.

There have been many reviews on the total syntheses of peptide natural products, particularly regarding synthetic methodologies, but this review highlights the total syntheses of peptidic natural products that provide the synthetic entry to the key biosynthetic intermediates to reveal the biosynthetic pathways and determine the correct structures of unusual peptides.

## Total synthesis of surugamide B and identification of a new offloading cyclase family

**Fig. 1:**
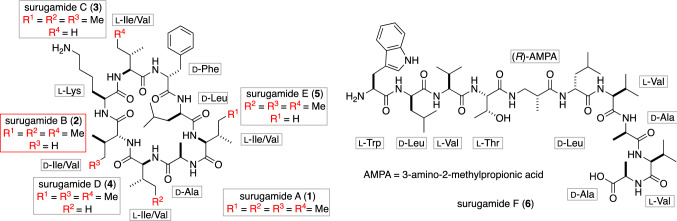
Proposed structures of surugamides A–E (**1**–**5**) and structures of surugamide F (**6**)

The cyclic octapeptides surugamides A–E (Fig. 1, **1–5**) were isolated from the marine actinomycete *Streptomyces* sp. JAMM992 [5]. In 2016, the biosynthetic gene cluster of surugamides was identified, which led to the discovery of the new linear decapeptide surugamide F (**6**) [6]. The cyclic peptides **1**–**5** show cathepsin B inhibitory activity, whereas the biological activity of the newly isolated **6** has not yet been evaluated. These cyclic and acyclic peptides are generated by a single gene cluster consisting of four successive non-ribosomal peptide synthetases (NRPSs), *surA*, *surB*, *surC*, and *surD*. Among these four NRPSs, *surB* and *surC* produce linear decapeptide **6**, which are sandwiched by the genes for **1**–**5** (*surA* and *surD*). Additionally, thioesterase (TE) domain, which terminates the peptide chain elongation by the hydrolysis/cyclization, was not identified at the terminus of this gene cluster [7]. Because of their unique NRPS genes, there has been much interest in the biological activity and also the biosynthetic mechanisms of the surugamides. However, the chemical synthesis for the structural confirmation and material supply of surugamides were not reported until 2018.

In 2018, Wakimoto and co-workers reported the first total synthesis of surugamide B (**2**). The development of an efficient synthetic route to **2** enabled the synthesis of the acyclic biosynthetic precursor, which led to the identification of a new offloading cyclase family [8].

The challenge in the synthesis of surugamides lies in the cyclization of the peptide, which is a long-standing problem for chemists [9]. The biggest obstacle in peptide chemical synthesis is the C_*α*_ epimerization, especially in the synthesis of cyclic peptides consisting of totally epimerizable *α*-amino acids, such as surugamides A–E (**1**–**5**). To minimize the side reactions including the isomerization, these cyclic peptides are usually synthesized with the optimization of the chemical reaction conditions (e.g., coupling reagent, solvent, and cyclization site) [10]. In contrast, the organisms generally produce cyclic peptides as stereochemically pure form, and surugamide B (**2**) is biosynthesized without detectable isomerization. Therefore, the synthesis of the cyclic peptide surugamide B was designed on the basis of the biosynthesis, similar to the preceding work on a cyclic depsipeptide theonellapeptolide Id [11].

**Fig. 2:**
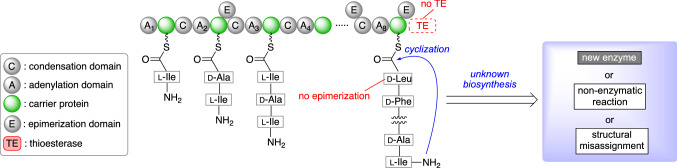
The biosynthesis of surugamide B (**2**)

The partly deciphered biosynthesis of **2** is illustrated in Fig. 2. However, the cyclization mechanism of **2** had not yet been elucidated due to the lack of TE domain. Wakimoto and co-workers identified the start and end points of the peptide synthesis. Namely, the biosynthesis of the linear peptide is initiated by the substrate-specific recognition of l-Ile by the first A domain (A_1_) and finishes with the attachment of l-Leu and the stereochemical inversion to d-Leu by the terminal E domain. Then, intramolecular amidation between l-Ile and d-Leu gives **2** without epimerization at the C-terminus.

**Fig. 3:**
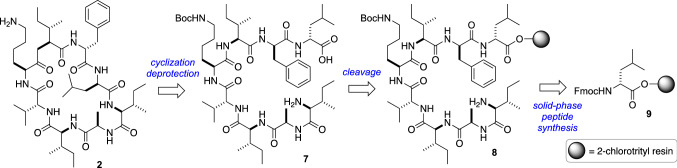
Retrosynthetic analysis of surugamide B (**2**)

Wakimoto’s plan for the total synthesis is summarized in Fig. 3. Using the genomic analysis, the peptide **2** was retrosynthetically linearized to **7**. The cyclization site was definitively determined from the genome analysis. The linear peptide **7** was then synthesized by the solid-phase peptide synthesis [12].

**Fig. 4:**
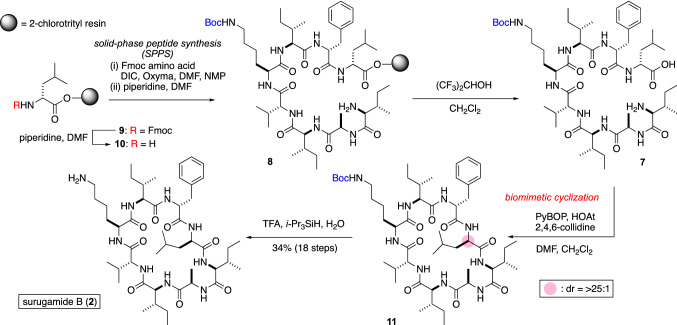
Total synthesis of surugamide B (**2**)

The synthesis of **2** commenced with the treatment of Fmoc-d-Leu loaded 2-chlorotrityl resin (**9**) with piperidine to liberate the amine **10**. Then, seven cycles of DIC/Oxyma [13]-mediated amide coupling and N_*α*_-deprotection were applied to **10**, leading to **8**. The cleavage of **8** from the resin was realized by the use of (CF_3_)_2_CHOH–CH_2_Cl_2_ (3:7) without the deprotection of the side-chain. The liberated acyclic peptide was then biomimetically cyclized using PyBOP [14]/HOAt [15] as coupling reagents to give cyclic peptide **11**. Finally, treatment of **11** with TFA-^*i*^Pr_3_SiH-H_2_O (95:2.5:2.5) afforded **2** with trace isomerization (*dr* =  > 25:1). After ODS-HPLC purification, **2** was obtained in 34% yield in 18 steps. The average yield per step in the total synthesis was 94%, which also supported the efficiency of the genome analysis-guided chemical synthesis (Fig. 4).

**Fig. 5:**
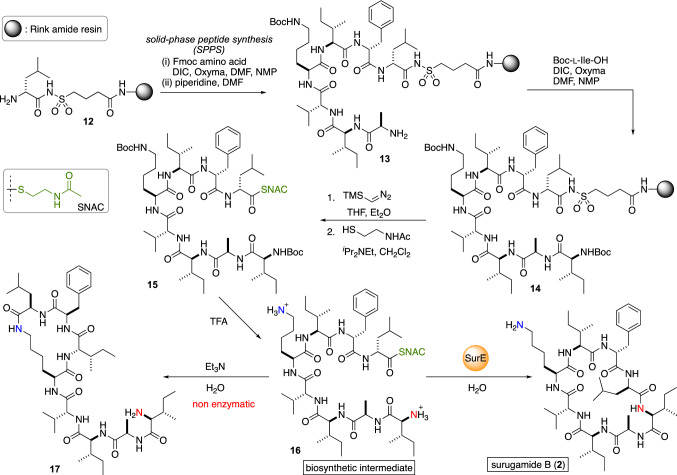
Investigation of the macrocyclization with chemically synthesized biosynthetic intermediate **16**

Having developed the biomimetic entry to **2**, Wakimoto and co-workers then elucidated the biosynthetic mechanism of surugamides. Considering the lack of a TE domain in the NRPS module of surugamide B, the non-enzymatic cyclizaition of the linear biosynthetic precursor was first examined. To investigate the cyclization mechanism, the *N*-acetylcysteamine (SNAC) thioester **16** was chemically synthesized as a mimic of the peptidyl carrier protein-bound peptide (Fig. 5) [16]. The resin-bound peptide **14** was synthesized in the same manner as the total synthesis of **2** utilizing the safety-catch linker strategy [17]. The sulfonamide of **14** was activated by TMS-diazomethane, and then reacted with SNAC to give thioester **15**. Then, the Boc group of **15** was removed by the action of TFA to deliver the linear biosynthetic precursor **16**.

Initially, a non-enzymatic cyclization of the **16** was performed in the presence of Et_3_N. However, the hydrolysis of the thioester was faster than the cyclization, and a considerable amount of isopeptide **17** was detected. These data strongly suggested that the head-to-side chain cyclization is more favorable in the non-enzymatic conditions, and an as yet unidentified peptide thioester cyclase is required for the head-to-tail macrocyclization in the later stage of the biosynthesis of **2**. The candidate genes for the off-loading enzyme would be SurE, which is encoded just upstream of SurA. The enzyme exhibits sequential similarity with penicillin-binding protein (PBP), which is a group of enzymes responsible for the transpeptidation step in the biosynthesis of bacterial cell wall peptide glycan [18]. To analyze the function of SurE, the enzyme was cloned into the pET-28a vector and expressed in the *E. coli* BL21 as a His-tagged protein, and then purified. When the recombinant SurE was mixed with **16**, the linear precursor **16** was efficiently transformed into **2** without any detectable by-products. These results strongly indicated that SurE plays a role in both chain termination and macrocyclization in the biosynthesis of surugamides A–E (**1**–**5**).

Although this section focuses on the chemical synthesis of surugamide B (**2**) and its biosynthetic precursors, Wakimoto and co-workers also reported the mechanistic studies of SurE after accomplishment of the total synthesis [19, 20]. Furthermore, the database search identified several homologues of SurE in the biosynthetic gene cluster of NRP such as mannopeptimycins [21], desotamides [22], ulleungmycins [23], and noursamycins [24]. Thus the total synthesis of a peptide natural product had opened an avenue for the discovery and the detailed functional analysis of a new cyclase family in the NRPSs.

## Total synthesis and structural revision of surugamide A

**Fig. 6:**
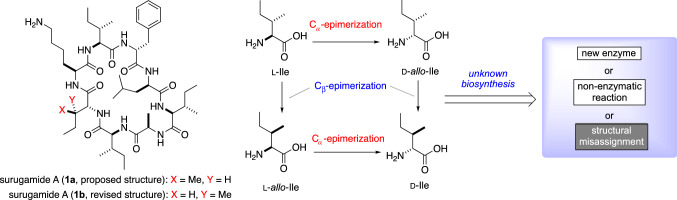
The structure of surugamide A (**1**) and the unknown nature of its biosynthesis

Another structural feature of surugamides is the d-Ile residue present in **1** and **3**–**5** (Fig. 1, **2** is a derivative with d-Val at this position). d-Ile is the enantiomer of l-Ile with the epimerization at both C_*α*_-, and C_*β*_-positions. The epimerization at the C_*α*_-position is commonly observed in the non-ribosomal peptide (NRP), and the C_*α*_-position of Ile is likely to be epimerized by the E-domain in the case of surugamides biosynthesis. However, the biosynthetic mechanism for the epimerization at the C_*β*_-position is obscure (Fig. 6).

**Fig. 7:**
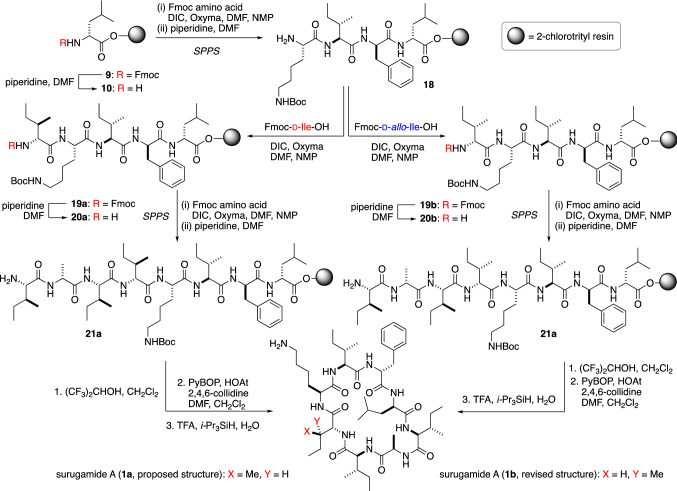
Total synthesis and structural revision of surugamide A (**1**)

The epimerization at the C_*β*_-position is very rare in nature [22, 25, 26]. Therefore, before launching an investigation into the biosynthesis of d-Ile residue, Wakimoto and co-workers first synthesized d-Ile-containing peptide **1a** to confirm the existence of d-Ile in **1** by taking advantage of the established route for the total synthesis of **2** (vide supra, Fig. 3). However, synthesized **1a** was not identical to natural **1** in the HPLC experiments, therefore, the reported structures of cyclic octapeptide surugamides **1** and **3**–**5** needed to be corrected. To determine the structure of **1**, solid phase peptide synthesis of d-*allo*-Ile-containing **1b** was also conducted (Fig. 7) [27].

The Fmoc group of **9** was removed by the treatment with piperidine to afford **10**, then three rounds of DIC/Oxyma-mediated amidation and *N*_*α*_-deprotection with piperidine yielded resin-bound tetrapeptide **18**. The amine **18** was first conjugated to Fmoc-d-Ile-OH to afford **19a**, and a further three rounds of solid-phase peptide synthesis (SPPS) was performed to generate the resin-bound octapeptide (**21a**). Successive treatment of **21a** with (CF_3_)_2_CHOH cleaved the peptide from the resin, and the peptide was subsequently cyclized by using PyBOP and HOAt to give the cyclic peptide. Finally, the peptide was treated with TFA/*i*-Pr_3_SiH/H_2_O (95:2.5:2.5) to remove the Boc group, which resulted in the originally reported structure of surugamide A (**1a**). However, the HPLC analysis revealed that synthetic **1a** was not identical to natural surugamide A. To reveal the true structure of **1**, d-*allo*-Ile-containing peptide **1b** was synthesized in the same manner as **1a**. Expectedly, the HPLC analysis showed that **1b** and natural **1** were identical.

In the original structural elucidation [5], the configuration of Ile was identified by a subtle chromatographic difference of the 2,3,4,6-tetra-*O-*acetyl-β-d-glucopyranosyl isothiocyanate (GITC) derivatives [28, 29], resulting in the misassignment. In Wakimoto’s study, the synthetic efforts confirmed that **1** contains a d-*allo*-Ile residue instead of d-Ile. Because of the commonality of the biosynthetic pathway, the d-Ile residue previously identified in other derivatives such as **3**–**5** should also be corrected to d-*allo*-Ile.

This section highlights the problem that the reported unusual structures of new natural products, especially targets for biosynthetic studies, are sometimes misassignments. As is the case with non-peptidic natural products, chemical synthesis plays an indispensable role in structural confirmation [4].

## Total synthesis of thioamycolamide A toward understanding the thioether biosynthesis

**Fig. 8:**
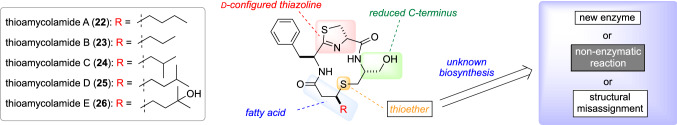
Structures of thioamycolamides A–E (**22**–**26**)

Organosulfur compounds are of interest in organic chemistry because of their particular bioactivities and biosynthses [30–32]. In 2020, Kakeya and co-workers reported the identification of the rare sulfur-containing cyclic lipopeptide thioamycolamide A (**22**, Fig. 8) along with the minor analogues thioamycolamides B–E (**23**–**26**) from the culture broth of *Amycolatopsis* sp. 26-4 [33]. The peptide **22** showed moderate inhibitory activities against several human cancer cell lines, and the chemical structure of **22** was established by a combination of spectroscopic analyses and the chemical synthesis of its partial structure. The cyclic skeletal structure of **22** contains a d-configured thiazoline, a thioether bridge, a fatty acid-side chain, and a reduced C-terminus.

While the highly modified structure of **22** is of interest to chemists, biosynthetically unusual structures are sometimes the result of structural misassignments. Therefore, the unprecedented structure of **22** needed to be confirmed by chemical synthesis. However, although several total syntheses of natural products that bear thiazoline rings [34, 35] or a reduced C-terminus [36] had been accomplished, the total synthesis of thioamycolamides was not reported until 2021.

**Fig. 9:**
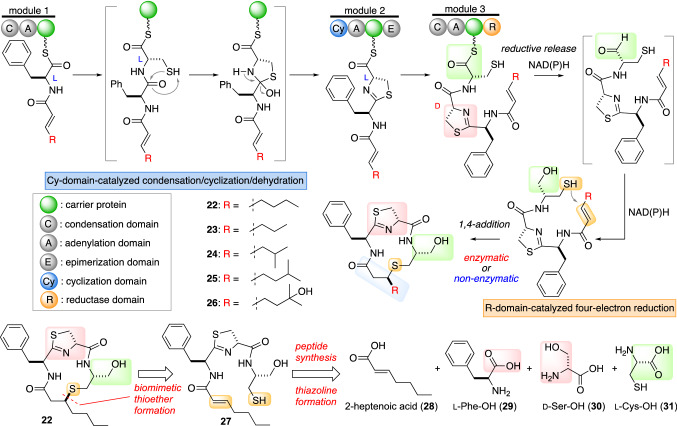
Proposed biosynthesis of thioamycolamides A–E (**22**–**26**) and a synthetic plan for **22** based on the proposed biosynthesis

In 2021, Kakeya and co-workers reported a concise total synthesis of **22**, which was designed based on the putative biosynthetic pathway, that corroborated the structure of **22** and the biosynthesis of the thioether bridge formation [37]. The retrosynthesis of **22** is summarized in Fig. 9. While the biosynthetic gene cluster has not been identified yet, the structural features of **22** suggest that this cytotoxin is assembled by an NRPS as illustrated in Fig. 9. Using this plausible biosynthesis as a guide, the macrolactam **22** was retrosynthetically acyclized to **27**. Then, the linear peptide **27** could be synthesized by the assembly of components **28**–**31** using isomerization-suppression procedures.

The challenge in the total synthesis of **22** had arose from the highly epimerizable nature of the thiazoline moiety [38–41]. Accordingly, the synthesis of the linear peptide **27** had to be constructed using limited reactions that would suppress the isomerization of the thiazoline.

**Fig. 10:**
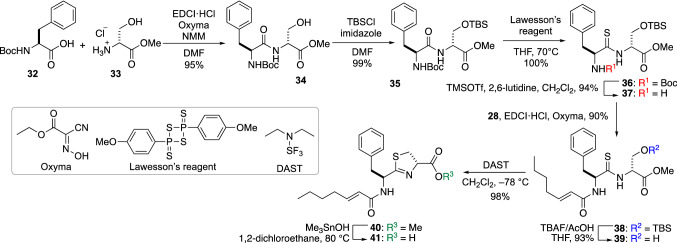
Synthesis of **41**

To overcome these problems, the total synthesis of **22** started from the chemical construction of the thiazoline moiety using Wipf’s cyclodehydration of *β*-thioamide [42] as the key reaction (Fig. 10). Initially, Boc-l-Phe-OH (**32**) was amidated with **33** to give **34**. The hydroxy function of **34** was protected by the TBS group to form **35**, and then dipeptide **35** was transformed into the corresponding thioamide **36** by the action of Lawesson’s reagent [43, 44]. To avoid the acid-promoted epimerization of the thiazoline, the fatty acid was attached ahead of thiazoline formation, i.e., the Boc group of **36** was selectively removed in the presence of the TBS group by the treatment with TMSOTf [45], and then the liberated amine **37** was condensed with acid **28**. At this stage, Kakeya and co-workers then attempted to transform a thioamide into thiazoline to prevent thiazolinone formation [46]. The undesired 1,4-additions to the *α,β*-unsaturated amide [47] of **38** also needed to be avoided. The TBS group of **38** was cleaved by TBAF/AcOH followed by successful cyclodehydration using diethylaminosulfur trifluoride (DAST) to provide thiazoline **40**. Finally, **40** was converted to carboxylic acid **41** by Nicolaou’s method [48] without appreciable thiazoline isomerization in the reaction. However, it should be noted that the epimerization of acid **41** was observed during silica gel column chromatography. Therefore, **41** had to be used immediately without chromatographic purification.

**Fig. 11:**
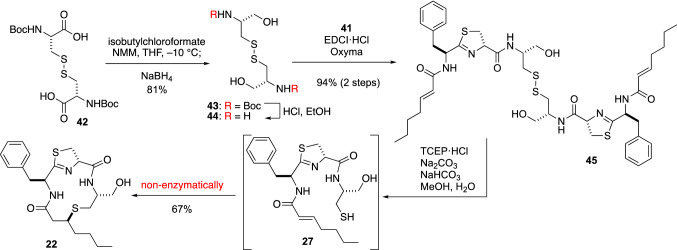
Total synthesis of thioamycolamide A (**22**)

With stereochemically pure **41** in hand, the total synthesis of **22** was accomplished by peptide chain elongation followed by bioinspired cyclization (Fig. 11). In Kakeya’s total synthesis, the dimeric cystine, from which the active thiol function can be liberated under mild reductive conditions, was used as an S-protected cysteine. Boc-l-cystine (**42**) was converted to the active ester, which was reduced in situ to alcohol **43** by NaBH_4_. The Boc group of **43** was removed by HCl, and then another building block **41** was attached to the N-terminus under EDCI·HCl/Oxyma conditions. Then, linear peptide **27** was successfully bridged by a thioether bond in pH 9 Na_2_CO_3_/NaHCO_3_ buffer containing TCEP·HCl [49] as a reductant. After reversed-phase ODS HPLC purification, **22** was obtained in 67% yield from **45**. This bioinspired total synthesis provided support for the proposal that the thioether bridge of thioamycolamides is stereoselectively biosynthesized by thio-Michael addition without any specific enzymes.

Although the NMR spectra of synthetic **22** agreed with the spectra of natural **22**, the spectroscopic data of peptides can vary depending on the solution conditions in the NMR tubes, and this often results in the structural misassignment of peptidic natural products [50–53]. An essential task at that stage was the structural confirmation of the chemically constructed **22** because the thio-Michael addition used in this synthesis may give **22** as a diastereomeric mixture due to the absence of a chiral catalyst. Notably, Kakeya and co-workers have recently developed several labeling reagents [54] inspired by the synthetic and structural studies on the peptidic natural products yaku’amides A and B [55, 56]. These highly sensitive labeling reagents are useful for the structural determination of peptides [57, 58] and identification of the scarce reactive natural products [59]. After the accomplished the total synthesis, the highly isomerizable structure of synthesized **22** was confirmed by utilizing their labeling reagents [60]. These experiments gave further support for the stereochemical purity of synthesized **22**, providing evidence that the thioether bridge of **22** can be formed by thio-Michael addition without biosynthetic enzymes.

This section has summarized the chemical synthesis of a biosynthetic intermediate that is spontaneously transformed into the natural product, shedding light on a non-enzymatic pathway in its biosynthesis.

## Conclusion

This review highlights the chemical syntheses of peptidic natural products that enabled synthetic entry to key biosynthetic intermediates, revealing the unique biosynthetic pathways and/or true structures of the natural products. The synthetic challenges involved in the construction of the cyclic octapeptide surugamide B (**2**) led to the discovery of a new cyclase family in the NRPSs; the synthesis of surugamide A (**1**) revealed the true structures of the surugamides, which have d-*allo*-Ile residues rather than d-Ile; and the biomimetic total synthesis of thioamycolamide A (**22**) demonstrated a non-enzymatic pathway for stereoselective thioether formation that may be present in its biosynthesis. Because biosynthetic studies on peptidic natural products are gaining importance in the identification of new natural products, the chemical synthesis of the key biosynthetic intermediates is becoming increasingly significant.

Although this review focuses on the chemical synthesis of non-ribosomal peptides (NRPs), the biosynthetic intermediates of ribosomal peptides [61] and non-peptidic natural products [62] can also be provided by the chemical synthesis, giving insights into the biosynthetic pathways of these natural products.
